# Feline Alimentary Lymphomas: Established Concepts and an Underexplored Molecular Landscape

**DOI:** 10.3390/cimb48020218

**Published:** 2026-02-16

**Authors:** Laura A. Szafron, Maciej Parys, Magdalena Parys, Lukasz M. Szafron

**Affiliations:** 1 Department of Experimental Oncology, Maria Sklodowska-Curie National Research Institute of Oncology, 02-781 Warsaw, Poland; laura.szafron@gmail.com; 2 Royal (Dick) School of Veterinary Studies & Roslin Institute, The University of Edinburgh, Edinburgh EH25 9RG, UK; maciej.parys@ed.ac.uk (M.P.); mparys2@ed.ac.uk (M.P.)

**Keywords:** alimentary lymphoma, classification and treatment, inflammatory malignancies, molecular diagnostics

## Abstract

Domestic cats are among the most popular companion animals worldwide, with steadily increasing ownership and life expectancy. Paradoxically, despite their high prevalence and shared environmental exposures with humans, cats remain markedly underrepresented in molecular oncology research. Cancer is a leading cause of feline mortality, and alimentary lymphoma (AL) has emerged as one of the most common feline malignancies, yet its molecular landscape remains poorly characterized. This review summarizes current knowledge on feline AL, including epidemiology, risk factors, classification schemes, diagnostic challenges, treatment outcomes, and survival, with particular emphasis on low-grade alimentary lymphoma (LGAL), the most prevalent subtype. We discuss the complex relationship between chronic inflammatory enteropathies and lymphoma, highlighting diagnostic ambiguities and the inflammatory–neoplastic continuum. Importantly, we provide a critical overview of existing genomic, transcriptomic, epigenomic, proteomic, and metabolomic studies in feline AL, revealing a striking paucity of high-throughput, multi-omics analyses based on clinical material. Recent advances in feline genome assembly and annotation offer new opportunities to address these gaps. Furthermore, we compare feline AL with its human gastrointestinal T-cell lymphoma counterparts, demonstrating substantial molecular homology across key oncogenic pathways, including JAK/STAT signaling. This comparative perspective underscores the potential of feline AL as a naturally occurring model for the human disease. We conclude that comprehensive molecular characterization of feline AL is urgently needed to improve diagnostics, prognostication, and targeted therapies, with likely translational benefits for both veterinary and human oncology. Aim: The goal of this review is to summarize the current knowledge on feline alimentary lymphoma, including its origin, risk, classification, treatment approaches, and especially molecular landscape, which still remains poorly investigated with modern high-throughput techniques.

## 1. Reverse Correlation Between Popularity and Molecular Insight: Why are Cats so Poorly Investigated?

Cats are one of the most popular pets in households all over the world. Every year, more people are becoming cat owners. In 2024, a 23% increase in cat ownership was observed. Also, an increasing number of owners are expanding their households to accommodate multiple cats [1]. The average lifespan of pet cats has also risen in the last decade. However, there are some discrepancies as to the life expectancy. The longest study encompassing about 3000 cats and 30 years of observations (years 1989–2019) showed that the median age of death equaled 9.07 years. There were no sex and racial biases (approx. 45.5% females and 54.5% males, and over 84% of cases were mixed-bred cats) [2]. However, the value of median lifespan (<10 years) was probably underestimated in the first few years of observations, as the median life expectancy of cats increased rapidly in the past decade [3]. A paper from 2015, which encompassed newer data (years 2009–2012) and over 2800 cats (a similar number of subjects as in the above-mentioned study performed in the USA), stated that the median lifespan of crossbred cats in England was 14 years [4]. However, the newest study from 2024 (comprising data from years 2019–2021, and over 7900 felines in the UK) showed that the life expectancy of crossbred cats was about 12 years [5]. Not being purebred is overall associated with increased longevity, just as having a lower body weight and being neutered [2]. According to another study, a 13.69% increase in life expectancy for mixed-bred cats was observed, which was significantly higher in 2019 than in 2013 [3].

Despite a longer lifespan due to, e.g., better healthcare, cancer is still one of the main factors affecting domestic cats’ mortality. A long-tem analysis performed by Kent et al. showed that cancer was the most common pathophysiological cause of death (35.81%), as it was identified in 41.3% of cats [2]. Additionally, studies indicate that most tumors in those animals are malignant. Unfortunately, feline cancers have not been as thoroughly investigated as those in dogs or, especially, in humans. The key areas of clinico-molecular research in many cancer types, such as feline methylome, transcriptome, and genomic variants analyses, remain poorly understood or entirely unexplored. One of the explanations may be the insufficient characterization of the feline (onco)genome, and for such analyses, the genome has to be well annotated. The newest version of the cat genome’s assembly (F.catus_Fca126_mat1.0) superseded the version Felis_catus_9.0, which had long gap-free segments and improvements in many genomic features (defined pseudogenes, lncRNAs, and novel genes added) compared to earlier assemblies of the feline genome [6,7].

Therefore, further extensive studies of these fields are of paramount importance, as the better characterization of the feline cancer genome holds great promise for the development of new diagnostic tools, prognostic indicators, and more targeted therapies. Given that domestic cats are subjected to similar environmental risk factors as humans, and additionally have shorter lifespans [8], such research could likely help understand and treat not only feline but also human cancers.

## 2. Lymphoma as One of the Most Prevalent Types of Feline Cancer—Risk Factors and Classification

### 2.1. Lymphoma Types and Risk Factors

Lymphoma, along with mammary cancer, squamous cell carcinoma, and sarcoma, is at the top of the feline cancer list [8]. A report from December 2024 even claims that lymphoma is the most common type of feline cancer [9]. Despite its prevalence, limited research has been conducted to understand the genetic landscape of feline lymphoma overall. Several of these studies have involved the use of feline lymphoma cell lines, and only a handful of studies utilized lymphoma tissues from cats. Summarized molecular analyses (including genetic translocations, expression analyses, immunophenotyping, and methylation patterns) performed on cell lines and clinical material obtained from lymphomas of different types are presented in the articles by Ludwig et al. [7] and Lin et al. [8]. The main types of feline lymphomas are mediastinal, multicentric, extranodal (e.g., renal), and gastrointestinal (GI, or alimentary, AL), with the last one being the most prevalent [10]. Among 163 cats with lymphoma, researchers found over half (52%) to have alimentary lymphoma [11]. In contrast to AL, other types of lymphomas are frequently associated with feline leukemia virus (FeLV) infections. For example, 80–90% of thymic lymphoma cats were positive for the FeLV antigen, whereas AL showed the lowest association with FeLV antigenemia, detected in only 0–12% of cases [10]. Of note, they also stated that lymphomas from FeLV antigen-positive cats are significantly more likely to be of T-cell immunophenotype than those found in FeLV antigen-negative cats. Moreover, an interesting observation was made for different FeLV forms. In several studies, the levels of FeLV antigen and the corresponding proviral DNA did not seem to be correlated. Also, approximately equal numbers of T- and B-cell lymphomas were provirus-positive [7,8,10]. Following oronasal exposure, the FeLV virus initially replicates in the oral lymphoid tissues before spreading through the peripheral blood via infected monocytes and lymphocytes [12]. It is unknown why the FeLV proviral form is sometimes unable to be activated and therefore expressed. One of the explanations may involve the TRIM25 protein. The ectopic expression of feline TRIM25 in HEK293T cells was correlated with reduced viral protein levels, leading to the inhibition of FeLV release [13]. In humans, TRIM25 regulates antiviral defense by modulating antiviral proteins and virus-related components involved in innate immunity, including, e.g., retinoic acid-inducible gene I (RIG-I). The conserved RING domain of TRIM25 interacts with RIG-I and ubiquitinates the CARD domain of RIG-I, which enhances the production of type I interferon (IFNα and IFNβ) [14]. This is an important mechanism, as type I interferons are effective agents in FeLV+ cats’ treatment. In the case of FeLV+ cats treated with recombinant human IFNα, it was demonstrated that, at the end of the treatment, 59.1% of cats had a decreased proviral load compared to the beginning of the treatment [15]. In humans, overexpression of TRIM proteins (including TRIM25) was shown to be associated with the development of different cancers, including lymphoma [16]. All these findings suggest that feline alimentary lymphoma may have a higher level of TRIM25 protein on its surface compared to other lymphoma types. However, further studies are necessary to verify this hypothesis.

Other factors that affect the formation of ALs are age, the presence of feline immunodeficiency virus (FIV), which correlates positively with the development of AL, exposure to tobacco smoke, and chronic inflammation (induced, e.g., by particular bacterial species like *Helicobacter* spp.) [10,17]. As for the diet, there are practically no studies showing the direct influence of particular nutrients on the development of AL; however, there are many papers concerning this question (and the impact of, e.g., hydrolyzed protein, omega-3 acids, refined carbohydrates) in the context of intestinal inflammation and its effect on the feline gut microbiome [18,19,20,21,22].

### 2.2. Alimentary Lymphoma Classification

ALs can also be defined according to the phenotype (T- or B-cell origin), histological grade (low, intermediate, or high), or cell size/morphology (small or large) of the neoplastic lymphocytes [23]. ALs may arise from both T- and B-cells. However, most of them are of T-cell origin [10]. A study performed on tissue sections from 50 AL cases, stained with hematoxylin and eosin, phosphotungstic acid hematoxylin, anti-CD3 (T-cell marker), anti-CD79a (B-cell marker), and anti-BLA.36 (B-cell marker), showed that T-cell lymphoma is mainly found in the small intestine, while in the large intestine and stomach, B-cell lymphomas prevail [24]. In the work by Wolsefberger et al. [25], performed on 61 cats, the predominance of B-cell lymphomas was also observed in the stomach (above 60% of cases), while the remaining samples exhibited a T-cell phenotype. In the small intestine, 90% of tumors were of T-cell origin. There was also one T-cell type tumor in the large intestine. At the ileo-cecocolic junction, lymphomas showed a B-cell phenotype. In the mesenteric lymph nodes, 90% of lymphomas had the T-cell phenotype. Wrapping up, a lymphoma phenotype was significantly associated with the tumor location.

Previously, using the National Cancer Institute Working Formulation (NCIWF) and the Revised European–American Lymphoma/World Health Organization (REAL/WHO) schemes, based on a grade and morphology, three main types of AL were defined: low-grade AL (LGAL also frequently defined as small-cell lymphoma (SCL); >90% of T-cell origin), high-/intermediate-grade AL (HGAL/IGAL; mainly composed of large cells of B- or T-cell origin) and large granular lymphocyte lymphoma (LGLL or LGL; >90% of T-cell origin). LGAL and IGAL/HGAL were considered to be distinct entities compared to LGLL. By contrast, IGAL and HGAL were pooled together because of similar clinical features. It is worth indicating that LGLL could be of any grade. A good and concise summary of this classification is presented in Table 1 in the paper by Barrs et al. [10].

For human T-cell lymphomas, the WHO classification (from 2008 revised in 2017 [26,27]) categorizes intestinal T-cell lymphomas into four distinct entities: Enteropathy-associated T-cell lymphoma (EATL, known also as EATL type I), monomorphic epitheliotropic intestinal T-cell lymphoma (MEITL, also known as EATL type II), intestinal T-cell lymphoma, not otherwise specified (ITCL-NOS) and indolent T-cell lymphoproliferative disorder of the gastrointestinal tract (GI-TLPD) [27]. As for cats, the classification system for this disease has been defined by Moore et al. and distinguishes two entities: “mucosal lymphomas” in which lymphocyte infiltrate is confined to the epithelium, are usually of a low grade, and are predominantly composed of small T-cells. They were also found to match the WHO entity EATL type II [23,28]. Nevertheless, a newer study by Freiche et al. [29] suggests that LGAL (referred to as low-grade intestinal T-cell lymphoma (LGITL) in this work), instead of being similar to MEITL (hallmarks of which, such as medium-sized tumor cells, CD56 positivity, and constant epitheliotropism, are not observed in feline LGAL), is rather a counterpart of human GI-TLPD. The second entity is “transmural lymphomas”, which are most frequently of high grade, and are composed of large or small cells of B- or T-cell type [10,23,28]. LGLL, which is an entity on its own, was also suggested to be classified as a transmural lymphoma and a subset of EATL type I [25,28]. The studies performed by Wolfesberger et al. [25,30], which are based on the new veterinary WHO classification, defined a few distinct subtypes of cat ALs. It is noteworthy that in the mentioned study, the T-cell lymphomas constituted circa 80% of cases. The authors additionally compared feline ALs to human counterparts, finding an overall classification consensus between these two species. Conversely, for EATL type I (or EATL), a quite low consensus (in over 60% of samples) was found, mostly due to the fact that cat counterparts did not meet all the criteria for human EATL type I. Hence, the human pathologist assigned the incompatible feline EATL type I mostly to the heterogeneous group of peripheral T-cell lymphomas, not otherwise specified (PTCL-NOS), or EATL type II. Despite Freiche et al. [29] suggesting that EATL type II/MEITL is not the counterpart of feline EATL II, Wolfesberger et al. [25] observed a 90% consensus for EATL II between feline and human lymphomas (based mainly on immunohistochemical staining). Other feline ALs in this classification included Diffuse Large B-Cell Lymphoma (DLBCL), T-cell-rich B-cell lymphoma (TCRBCL), T-cell anaplastic large-cell lymphoma (TALCL), and extranodal marginal zone lymphoma of mucosa-associated lymphoid tissue (MALT). For DLBCL, TALCL, and MALT, a 100% consensus between feline and human lymphomas was found. One cat, diagnosed with TCRBCL by the veterinary pathologist, was reclassified as DLBCL-afflicted by the human pathologist. In veterinary medicine, TCRBCL is classified as a variant of DLBCL, consisting of sheets of small reactive T-cells, which usually predominate, and large neoplastic B-cells. However, if the B-cells are arranged in groups, the lymphoma should be diagnosed as DLBCL. The last type, feline PTCL-NOS, is compatible with human ITCL-NOS (formerly also known as PTCL-NOS) [25,30]. In the publication by Kehl et al. [31], the authors also concisely summarized the new WHO veterinary classification from 2017, which first classified ALs according to the size of the neoplastic cells and the immunophenotype into MALT, SCL, LGL, and multicentric lymphomas, which do not affect the gastrointestinal tract alone. However, they also stated that modified nomenclatures described in [32] and the above-mentioned [25] are available; however, the authors suggest that such classifications, especially when concerning translation between human and feline systems, may have some limitations [31].

Table 1 shows the summary of the classification of feline lymphomas and their human counterparts.

**Table 1:** The summary of feline lymphoma classification along with their human counterparts.

General Classification Based on the NCIWF and REAL/WHO Schemes (According to Lymphoma Grade and Immunophenotype/Morphology) (Barrs et al. [10])	
Low-grade AL: LGAL (other terms—SCL, EATL II, LGITL): mainly T-cells	
Intermediate/high-grade AL: IGAL/HGAL: B- or T-cells	
Large granular lymphocyte lymphoma: LGLL (other terms—LGL): mainly T-cells	
**Feline AL types**	**Human counterparts**
**Moore et al. [28] (based on human WHO classification from 2008)**	
Mucosal lymphomas (mainly of small-cell type and of low grade)	EATL II
Transmural lymphomas (mainly large cells, typically of high grade. LGLL is also a big subset of transmural lymphomas)	EATL I
DLBCL	?
Veterinary WHO classification (2016) presented in Wolfesberger [25,30] * (human WHO classification from 2017)	
EATL II	EATL II (MEITL—newer designation). However, Freiche et al. [29] proposed GI-TLPD as a human counterpart
EATL I (with LGLL as a subtype)	Not all cases were similar to human EATL I; incompatible samples classified as PTCL-NOS/ITCL-NOS or EATL II
PTCL-NOS	ITCL-NOS
DLBCL	DLBCL
MALT	MALT
TALCL	TALCL
TCRBCL	DLBCL
**Veterinary WHO classification (2016) presented by Kehl et al. [31]**	
SCL	-
MALT	-
LGL	-
Multicentric lymphomas	-
**Veterinary WHO classification (2016) presented by Munday et al. [32]**	
DLBCL	-
MALT	-
EATL I	-
EATL II	-
LGL	-

(-) Authors did not raise this question/did not compare those entities. * Wolfesberger [30] also proposed that T-cell lymphomas with CD3−, granzyme B+, CD5−, CD4−, CD8+, CD56+ phenotype could be assigned as NK-cell lymphomas in cats (this entity is not designated as a specific subtype in the current WHO classification for animals, but is specified in the current human WHO classification).

From among all the aforementioned feline lymphoma entities, the most frequent is LGAL. The morbidity due to this disease has increased over the last few years, and now, it is the most frequent digestive neoplasm in cats, diagnosed in 60 to 75% of gastrointestinal lymphoma cases [23].

## 3. Inflammatory Bowel Malignancies vs. Lymphoma—Differences and Similarities

Chronic enteropathy (CE) is a common condition in cats, particularly among older individuals, and its prevalence has increased over the past two decades. There is still a question of terminology for enteropathies, and the description of CE in cats used in the literature varies. Overall, chronic inflammatory enteropathy (CIE) and small-cell alimentary lymphoma (SCL or LGAL) are defined as feline chronic enteropathy (FCE or CE). Additionally, the most common histologic type of feline CIE is lymphocytic plasmacytic enteritis (LPE), with the inflammatory infiltrate localized to the lamina propria (and in some cases, the intestinal epithelium) and often accompanied by architectural lesions of tissue [33]. Marsilio et al. [34] proposed the following definitions:

1. The chronic enteropathy term is reserved for cats with chronic (at least 3-week duration) signs of gastrointestinal disease where extra gastrointestinal, metabolic, and infectious causes have been ruled out. 2. LPE for inflammatory lesions occurring in the gastrointestinal tract of cats with CE that are dominated by lymphocytic infiltration in the lamina propria. 3. LGAL for lesions in the gastrointestinal tract of cats with CE, characterized by a monomorphic infiltration of the lamina propria or epithelium (or both) with small, mature, neoplastic (clonal) T lymphocytes.

The term inflammatory bowel disease (IBD) frequently appears in the literature, but its definitions are often vague. To clear things up, IBD is sometimes used interchangeably with CIE, as IBD in humans shares similarities with feline CIE. Additionally, both are chronic, idiopathic conditions with a relapsing course. Consistently with this definition, in some publications [35], IBD and CIE are classified in the same group. Other researchers define IBD as a unit encompassing a heterogeneous group of immune-mediated disorders of the digestive tract, commonly manifesting as chronic LPE affecting the small intestine [36,37].

**Figure 1:**
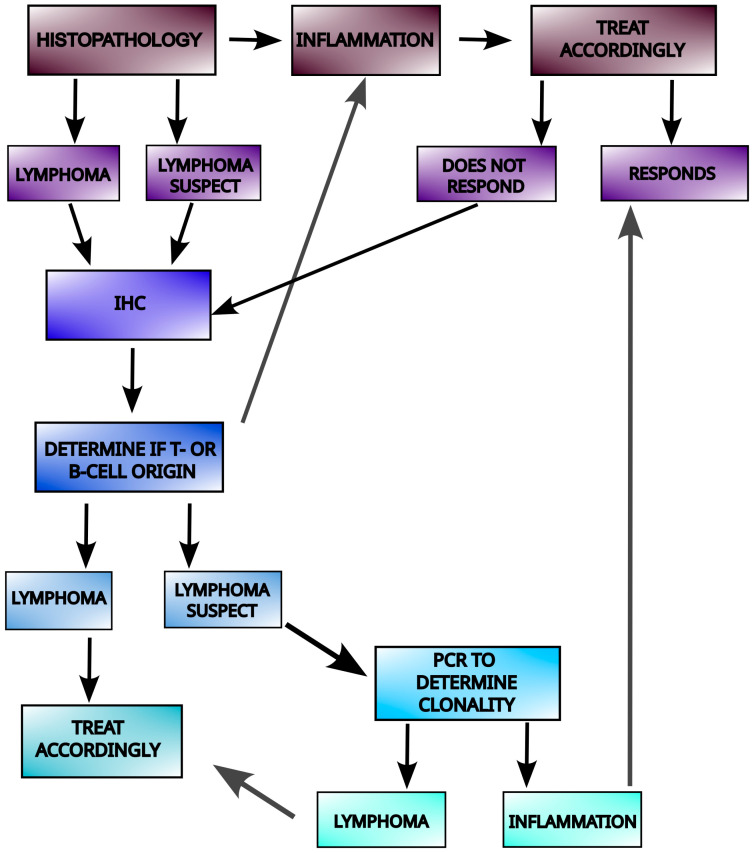
A diagnostic algorithm that utilizes histopathology assessment, IHC for CD3 and CD79, and PARR (PCR for Antigen Receptor Rearrangements) to differentiate IBD from EATL II in felines (adapted from Munday et al. [32]).

As those enteropathies share some similarities, a significant number of feline intestinal small-cell lymphomas (SCL/LGAL) are misdiagnosed as inflammation (IBD/CIE) when assessed by histology alone or combined with immunohistochemistry. This misdiagnosis may occur due to ambiguous changes in mucosal architecture or sparse lymphoid infiltration at the biopsy site. Additionally, SCL can arise from chronic inflammation, with transitional phases where monoclonal proliferation is detectable by PCR, though histological monomorphism (uniform appearance of cells) is not yet visible. However, lymphoid clonality does not always indicate neoplasia. Oligoclonal or monoclonal expansions of reactive lymphocytes with indistinguishable gene rearrangements may also occur in chronic infections, including IBD, potentially causing false-positive diagnoses of cancer [35,37]. The algorithm for LGAL/IBD discrimination (based on Munday et al. [32]) is presented in Figure 1.

The last question concerning similarities and differences between distinct CE entities is the composition of the gut microbiome. It was shown that cats with CE (including IBD and SCL) had reduced overall bacterial diversity when compared to healthy cats; however, no significant difference was found between IBD and SCL groups. Overall, in cats with CE, decreases in beneficial *Firmicutes*, *Actinobacteria* and *Bacteroidetes* and increases in unfavorable *Enterobacteriaceae* and *Streptococcaceae* were observed. Such a pattern resembles dysbiosis often seen in human IBD [38]. By contrast, other study performed on a very similar group revealed that there are significant differences between IBD and SCL, as higher levels of some bacteria species (*Fusobacterium* spp. and *Bacteroides* spp.) were present in SCL in some intestinal regions. Additionally, there was a correlation between the presence of *Fusobacterium* and some inflammatory markers (e.g., NF-kB) [39].

## 4. AL Diagnostics, Treatment, and Survival

Diagnostic evaluation of cats with AL typically involves abdominal ultrasonography and ultrasound-guided fine-needle aspiration, which often supports lymphoma suspicion. However, definitive subtype classification requires histopathological analysis of high-quality tissue samples, including hematoxylin and eosin (HE) staining and immunohistochemistry (IHC) for specific markers (detailed below). When IHC is unavailable, PCR-based clonality tests (PCR for Antigen Receptor Rearrangements, PARR), assessing rearrangements in gene segments for immunoglobulin heavy/light chains, and for T-cell receptors, may be used [23,25].

The treatment methods for the three main subtypes of AL are well summarized in Table 1, presented by Barrs et al. [10]. A multi-agent CHOP-based (cyclophosphamide, hydroxydaunorubicin (doxorubicin), Oncovin (vincristine), prednisolone) scheme is used for curing HGAL/IGAL as well as LGLL, while the treatment of LGAL (SCL) requires prednisolone and chlorambucil. The median survival for LGAL (EATL II) is quite high and equals 19–29 months. For HGAL/IGAL (EATL I or PTCL-NOS according to the newest classification), the patients survive 7–10 months. The lowest median survival is for LGLL (EATL I subtype) and equals 17 days [10]. By contrast, Wolfesberger [25] states that the median survival time for EATL I (large-cell lymphomas overall) equals only 1.5 months (much less than the median survival times presented in [10], for HGAL/IGAL and LGLL analyzed together), and for EATL II, such a median is 2.4 years (which is consistent with the median survival times presented by Barrs et al. [10]). The publication by Barrs et al. is slightly outdated; still, the treatment methods have not changed significantly over the years. In the study from 2019, by Wright et al. [40], it is shown that the current standard of LGAL (SCL) care involves combination therapy with glucocorticoids and chlorambucil. The researchers first referred to other publications, which stated that various dosing regimens might be applied (yielding response rates between 69% and 96%), treatment is generally well tolerated, with minimal reported toxicities, and the median survival times for SCL range from about 800 to 1300 days. However, according to the results obtained by Wright et al., the median survival time had a significantly smaller lower range limit (204–1267 days) [40]. For large-cell lymphoma (LCL; in the discussed study, this term comprises HGAL as well as LGLL), multi-agent chemotherapy regimens, often variations of CHOP, are recommended. Compared to other publications, where remission rates varied from 30% to 80%, with median survival times ranging from about 100 to 200 days (or longer in cats achieving complete remission), Wright et al. demonstrated that the median survival time range had a similar upper value, yet a noticeably smaller lower value (2–183 days) [40]. In another study encompassing cats with HGAL [41], the median overall survival time was much higher and equaled 417 days (range: 12–2962 days). However, no LGLL cases were included in this sample set.

The treatment of cat lymphomas, especially LGLL, still remains challenging. While multi-agent CHOP-based chemotherapy is used, clinical approaches vary widely in the literature, including surgery, CHOP protocols, lomustine, radiation therapy, corticosteroids, or no treatment. In spite of these efforts, cats with LGLL generally show minimal response, with a median survival time of only 57 days in treated cases. Generally speaking, effective treatment regimens for feline LGLL have not yet been well established [10,42].

The methods of treatment and the survival times for various lymphoma types are summarized in Table 2.

To sum up, considering that the detection and treatment methods of AL in cats are still not fully defined and reliable, the preparation of new diagnostic tests and more effective, less aggravating therapies, especially those targeting specific molecular markers, is of paramount importance.

## 5. Molecular Insight and Landscape of AL: An Update

Nowadays, high-throughput techniques (employing, e.g., next-generation sequencing, microarrays, or mass spectrometry) are widely used in human oncology. In recent years, most articles concerning feline ALs focused on clinical aspects (chemotherapy administration, response to treatment, clinicopathological findings, lymphoma classification, etc.), and there were practically no papers regarding the molecular features of those tumors. Therefore, as mentioned at the beginning, there is a very limited number of studies encompassing whole genome (or exome), methylome, transcriptome, proteome, and metabolome analyses in clinical material in cats.

A few more studies were performed for feline lymphoma cell lines. These are neatly summed up in Table 2, prepared by Ludwig et al. [7]. Unfortunately, even those molecular analyses concentrated on chromosomal rearrangements and only a few genes, mainly *TP53*, and methylation sites [7]. Additionally, according to the PubMed database, there were very few publications dealing with the molecular aspects of feline ALs after the year 2022.

### 5.1. Genome

One paper described *STAT5B* and *STAT3* gene statuses in cats with AL. A *STAT5B* mutation (c.1924A > C) was found in *EATL* type II. This mutation leads to the substitution of asparagine 642 with histidine (N642H), resulting in a gain-of-function (GOF) mutation, which leads to prolonged tyrosine phosphorylation, causing hyperactivation and the constitutively active STAT5B protein. By contrast, STAT3 exhibited strong activation in AL cells, despite the absence of detectable nucleotide changes linked to known activating mutations [43]. Interestingly, human and feline STAT5B and STAT3 protein sequences are 97.5% and 100% identical, respectively [43]. Therefore, one may presume that human and feline intestinal lymphomas share, at least in part, a common molecular background, which makes references to comparable studies in humans quite reasonable. Accordingly, the study from 2002 found the *NRAS* Q61K mutation in one case of feline alimentary lymphoma. This genetic aberration turned out to be of somatic origin, as blood cells of the same cat did not harbor this polymorphism [44]. Of note, the same genetic alteration is also frequently found in human malignancies [45,46].

As mentioned above, one of the genomic studies routinely performed for lymphomas is PARR. This clonality assay is used to distinguish neoplastic from inflammatory lymphoid cell populations. Lymphoid neoplasms represent monoclonal expansions of malignant cells, whereas inflammatory responses typically involve polyclonal lymphoid populations. These assays rely on PCR amplification, which targets the T-cell receptor gamma (*TCRG*) and immunoglobulin heavy (*IGH*) chain genes. PCR primers are designed to bind conserved regions flanking the hypervariable complementarity-determining regions (CDR3) of these genes [47].

### 5.2. Transcriptome

To date, transcriptome analyses have been more frequently reported for feline lymphoma in general than specifically for AL. One study aimed to determine the mRNA levels of MDR1 and COX2 in cats with IBD and LGAL, and to evaluate their correlation with clinical symptoms, histological severity, and the mutual correlation of expression patterns between these two genes. No correlations were observed between the feline CE activity index (quantitative indicator of inflammatory activity in cats affected by CE), histological grading for IBD, and between MDR1 and COX2 expression levels. MDR1 and COX2 gene expression levels were, however, increased in cats with LGAL compared to cats with IBD. Nevertheless, it was only a pilot study [48]. In another paper, FAS splice variants were analyzed in lymphoma cell lines and clinical material obtained from different types of feline lymphoma, including AL. Researchers discovered shorter FAS transcripts in this malignancy, which led to the production of Fas protein lacking the transmembrane domain, which likely impairs apoptosis [49]. Another study, involving 11 lymphomas (including intestinal), five carcinomas, and five control tissues, analyzed microRNA expression. The values of miRNA-20b were found to be up-regulated in all types of tumors, whereas miRNA-192 was up-regulated in carcinomas and B-cell lymphomas only. Unfortunately, this study was also just a pilot [31].

No other studies concerning RNA expression are present for feline AL. However, in feline IBD, the RNA expression coding for pro- and anti-inflammatory cytokines, such as IL-2, IL-4, IL-5, IL-6, IL-10, IL-12 (p35 and p40), IL-18, TNF-α, IFN-γ, and TGF-β, has been characterized, supporting the concept of the dysregulated intestinal immune environment. Given the proposed inflammatory-to-neoplastic continuum, such transcriptomic analyses may provide mechanistic insights into the progression from inflammatory state to LGAL [50].

### 5.3. Methylome

For feline AL, there are practically no methylation studies involving whole genome methylation analyses and clinical samples from a big cohort. As for cell lines, some methylation genome-wide studies were performed, but they were only preliminary, focused on a few genes, and carried out on cells derived from feline lymphomas of unspecified origin (the authors did not unambiguously define whether it was AL) [7]. Another study, utilizing not only cell lines but also concentrating on methylation statuses in clinical material originating from feline AL, revealed aberrations in *p16* (*CDKN2A*) in B-cell gastrointestinal lymphoma [51].

### 5.4. Proteome and Metabolome

Studies involving whole proteome/metabolome analyses (involving, e.g., mass spectrometry or chromatography) are also quite scarce in cats. There are only a few publications dealing with this topic. One is the analysis of serum proteome using mass spectrometry, in which the authors stated that thrombospondin-1 (THBS1) could be a good marker used in the diagnostics of feline CE (including LGAL) [52].

Another research employs histology-guided mass spectrometry for the differentiation of LPE from SCL (LGAL) in duodenal FFPE cat samples. This is a technique in which mass spectral profiles are acquired from each annotation site within a tissue sample, and are analyzed using machine learning algorithms to identify differences in peak intensities indicative of specific molecular features. However, in this study, the complete proteome was not analyzed, and no other broad, functional analyses (such as, e.g., gene ontology enrichment analysis) were conducted [53].

Most prevalent proteomic analyses among AL involve immunophenotyping using IHC or flow cytometry. One of the newest studies in this area by Wolfesberger et al. [30] revealed that neoplastic lymphoid cells are positive mainly for granzyme B and CD3, and to a lesser extent for the CD4, CD5, CD8, and CD56 antigens. The researchers also stressed the fact that complex immunophenotyping should be frequently used, as cytotoxic granules (containing granzyme B) were identified by histopathology only in 13% and by cytology in 47% of the cases. Without immunohistochemical labeling of granzyme B, the cytotoxic status would have been missed in 46% of the cytological and in 85% of the histopathological slides. All immunophenotypes are clearly presented in Table 3 from their article [30].

Another recent paper described detailed flow cytometric analyses of different types of feline AL and analyzed even more antigens. The following number of small intestinal lymphomas were investigated in the mentioned study: 10 EATL I, two EATL II, two PTCL, three DLBCL, and one DLBCL+EATL II. The most common small intestinal T-cell phenotype was CD3+CD21− CD4−CD8−CD18+ CD5−CD79−, found in 7/10 EATL I and one EATL II. The most frequent B-cell phenotype was CD3−CD21+ CD4−CD8−CD18+ CD5−CD79+, identified in 13/17 DLBCL and in the DLBCL+EATL II [54]. In the article by Ii et al. [55], who separated feline ALs based on their morphology (LCL and SCL), all cases were positive for CD3 and negative for CD79α, CD30, CD56, and FOXP3 antigens. In addition, LCLs were positive for T-cell intracellular antigen 1 (TIA1) in 100% of cases, for CD8 in almost 100% of cases, and granzyme B in over 40% of cases. SCLs were positive for TIA-2 (92%), CD8 (78%), and granzyme B only in 6% of cases (in contrast to high granzyme B expression presented in [30]). Moreover, TIA1- and granzyme B-positive neoplastic lymphocytes were predominantly observed in the mucosal epithelium, while the patients with such an immunophenotype had significantly shorter survival times, suggesting that mucosal epithelium infiltration by neoplastic cells with a cytotoxic immunophenotype is a negative prognostic factor [55]. Finally, as mentioned before, Freiche et al. immunophenotyped the expression of STAT proteins in feline LGAL (SCL) and observed that those cells were 100% positive for phosphorylated STAT5 and negative for phosphorylated STAT3 [29].

There are also some studies analyzing a single or a few proteins. For example, one study assessed the expression of S100A12 and S100A8/A9 calgranulin in the tissue biopsies in cats with bowel inflammation and AL [33]. It is worth mentioning that only minor differences in calgranulin expression were observed between healthy and diseased animals, with no significant differences between cats with intestinal inflammation and those with lymphoma. Nevertheless, significant correlations were observed between calgranulin-positive cells, microscopic inflammatory changes, and clinical disease severity. Overall, the authors concluded that calgranulins may contribute to both gastrointestinal lymphoma and inflammation, supporting the emerging hypothesis that these conditions may not be entirely distinct, but rather are part of a shared disease spectrum. The measurement of S100A12 was also performed in feces from cats with CE (IBD/CIE and LGAL). This research showed that the level of the above-mentioned protein is higher in cats with CE when compared to healthy controls, but does not differ significantly between CIE/IBD and LGAL [35].

Another research team, using IHC, demonstrated a significantly higher percentage of cells positively immunolabeled for Bcl-2 in cats with AL compared to those with IBD, which may potentially serve as a disease discriminator, as well as a drug target for venetoclax, which is a selective Bcl-2 inhibitor used in, e.g., human lymphomas [56,57].

The last study dealt with the level of serum amyloid A (SAA) in feline sera. The research unveiled that LGAL cases had a considerably higher SAA concentration when compared to HGAL at initial presentation. The SAA concentration at initial presentation was not a reliable prognostic or diagnostic marker for HGAL and showed no association with lymphoma grade or stage. Conversely, elevated SAA levels observed on day 56 appeared to represent a potential novel biomarker of poor prognosis, particularly in HGAL cats [58].

The analysis of the serum metabolome was also performed for CE. Several metabolites were found to be significantly different between cats with IBD compared to LGAL, including some sphingolipids, phosphatidylcholine 40:7, uridine, pinitol, 3,4-dihydroxybenzoic acid, and glucuronic acid. As mentioned earlier, the discrimination between IBD and LGAL is hard and tricky. Thus, such a study could have potentially paved the way for the creation of new diagnostic tests based on the aforementioned markers. Nevertheless, this study had many flaws and limitations. Foremost, the random forest model created by the authors was characterized by rather poor performance, with an accuracy of differentiation between IBD and LGAL of merely 60%. Another significant limitation of this study was not excluding dietary-responsive enteropathy in all cats, and not performing some fecal cultures for known pathogens. The authors also stated that some cases could have been misclassified due to the lack of a clear gold standard in the differentiation of IBD from LGAL. Unfortunately, the IHC stainings for, e.g., pSTAT3, pSTAT5, or Ki-67 (presented in [29]), were not performed by the authors, which could be particularly useful to further explore this issue [59].

A similar metabolomic study, which also included cats with chronic enteropathy (IBD and SCL), was conducted by Marsilio et al. [60]. They found that polyunsaturated fatty acids hold discriminatory potential in differentiating IBD from SCL. The metabolomic profiles of cats with CE closely resembled those observed in humans with similar conditions, showing significant alterations in metabolites associated with the tryptophan, arachidonic acid, and glutathione pathways. Several amino acids were found to be elevated in the feces of cats with CE. Metabolites related to glutathione metabolism (such as 2-hydroxybutyrate/2-hydroxyisobutyrate and gamma-glutamylglutamine), as well as arachidonate (an inflammatory mediator) and multiple sphingolipids, were significantly more abundant in CE-affected cats. Omega-3 polyunsaturated fatty acids, including eicosapentaenoate, were useful for distinguishing SCL from healthy controls, but not from IBD. In contrast, indole derivatives such as 2-oxindole-3-acetate and 5-hydroxyindoleacetate were significantly reduced in feces from CE cats. Of note, this study also had some limitations, such as the fact that food-responsive enteropathy was not ruled out in all participants, and fecal cultures for specific pathogens were not performed [60].

**Figure 2:**
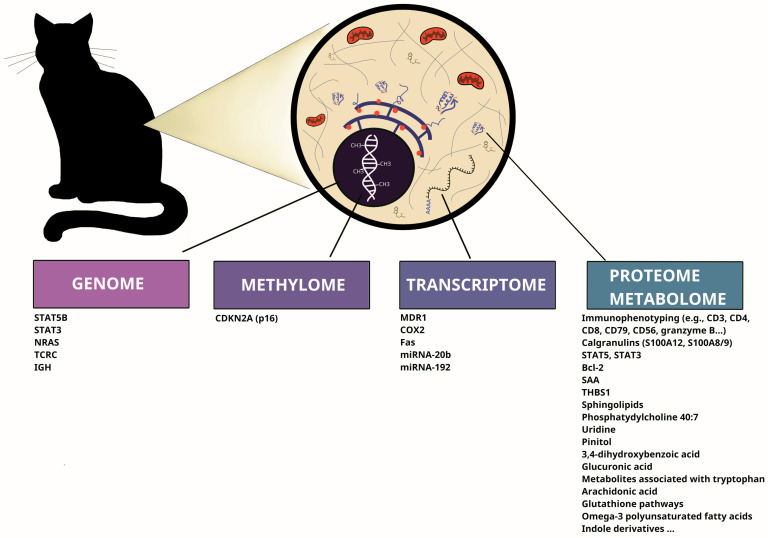
The graphical summary of various molecular biomarkers, identified for feline AL in different studies.

The graphical summary of various molecular biomarkers, identified for feline AL by different research groups (at the genome, methylome, transcriptome, proteome, and metabolome levels), is shown in Figure 2. Additionally, Table 3 contains the number of cases/samples in each molecular study discussed above.

**Table 3:** The list of various molecular biomarkers identified for feline AL in different studies.

Analyzed Molecular Target(s)	Material (Group)
**Genome**	
*STAT5B* (*N642H*), *STAT3* [43]	Clinical retrospective material (FFPE tissue blocks, T-cell AL, *n* = 42)
*NRAS* Q61K [44]	Tumor samples from 15 feline patients with lymphomas (variant in *NRAS* found in cat with small intestine lymphoma)
**Transcriptome**	
*MDR1* and *COX2* [48]	Biopsy samples from 20 cats with IBD and 9 cats with LGAL
*FAS* [49]	5 feline lymphoma cell lines Primary tumor tissues (11 cats with lymphoma, including AL. The number of cats with AL was not specified)
miRNA-20b, miRNA-192 [31]	Samples selected based on big dataset analysis. For miRNA expression, large-cell B-cell lymphomas (*n* = 6), small-cell T-cell lymphomas (*n* = 5) and tubular carcinomas (*n* = 5) of the small intestine were randomly chosen (FFPE tissue)
**Methylome**	
*CDKN2A* (*p16*) [51]	5 lymphoma cell lines Fresh frozen primary tumor samples from 39 cats with lymphoid neoplastic diseases (including 12 with gastrointestinal lymphoma (6 of B-cell origin))
**Proteome**	
**Whole/partial proteome analysis**	
Identification of THBS-1 as a good marker of CE diagnostics [52]	Multicenter study (3 veterinary hospitals). Serum from cats with gastrointestinal diseases (12 cats with CE, including LGAL)
Histology-guided mass spectrometry [53]	FFPE duodenal cat samples (41 with LPE and 52 with SCL)
**Immunophenotyping (using IHC and/or flow cytometry)**	
IHC combined with flow cytometry (CD3, CD4, CD5, CD8, CD20, CD21, CD56, CD57, granzyme B) [30]	Samples (obtained by laparoscopic resection) from 15 cats with non-B-cell AL
Flow cytometry (CD3, CD21, CD4, CD8, CD18, CD5, CD79) [54]	Material from gastrointestinal mass (32 cats with large-cell lymphomas of T and B type. Most of patients suffered from intestinal lymphoma)
IHC (CD3, CD8, TIA1, granzyme B, CD30, FOXP3, Ki-67, CD56, CD79) [55]	Tissue samples from 50 cats with intestinal T-cell lymphoma (large and small lymphoid cells)
IHC (CD3, CD4, CD8, CD20, CD56, Ki67, phosphorylated STAT5B and STAT3) [29]	Gastrointestinal biopsies from 22 cats with LGAL (LGITL)
**Analysis of single/few protein(s)**	
S100A12 and S100A8/A9 [33]	Tissue samples from cats with CIE (*n* = 16) and SCL (*n* = 8)
S100A12 [35]	Fecal samples from 49 cats with CE (19 with IBD and 30 with AL)
Bcl-2 [56]	FFPE tissue blocks from 55 cats with CE (8 cats with IBD and 47 cats with AL)
SAA [58]	Serum samples from 39 cats with AL (HGAL: *n* = 17, LGAL: *n* = 22)
**Metabolome**	
All significantly altered metabolites (in cats with CE) are presented in Table 3 [59]	Serum from 28 cats with CE (14 with IBD and 14 with LGITL/LGAL)
All significantly altered metabolites (in cats with CE) are presented in Table 2 [60]	Fecal samples from 22 cats with CE (11 with IBD and 11 with SCL)

## 6. Human AL Counterparts—What Impact May They Have on Feline AL Diagnostics and Treatment?

Despite the vivid process of reclassification and some discrepancies in the terminology used for human and feline alimentary lymphomas (Table 1), there is an evident resemblance between those entities in the above-mentioned species. Therefore, a better understanding of the human molecular landscape in AL may be of particular use in cats (and vice versa). The detailed comparison of human and feline ALs has already been made in the publication by Wolfesberger et al. [25].

In human lymphoma counterparts, specifically primary gastrointestinal T-cell and Natural Killer-cell lymphomas (GI-TNKLs), which include entities such as MEITL and EATL, numerous genetic alterations have been identified. For EATL, recurrent gains in chromosome 9q, particularly within the 9q33-34 region encompassing the *NOTCH1* and *ABL* genes, have been reported. Overexpression of *NOTCH1*, potentially driven by activation under the T-cell receptor promoter, appears to contribute to lymphomagenesis. Additionally, amplification of this region may also involve *CDK9*, a gene encoding a cyclin-dependent kinase that plays a crucial role in cell cycle regulation.

By contrast, in MEITL, frequent mutations have been described in *STAT5B*, *STAT3*, *GNAI2*, and *SETD2*, genes implicated in signal transduction and epigenetic regulation. Other genes recurrently mutated across GI-TNKLs include *TET2*, *JAK1*, *JAK3*, *KCNB2*, *TP53*, *CSMD2*, *CSMD3*, *IGSF10*, and *FSIP2*, suggesting a complex mutational landscape that influences various cellular pathways such as DNA methylation, cell signaling, and tumor suppression [27,61,62].

We used the Uniprot database [63] and the Expasy SIM tool [64] to assess the similarity of sequences between human and cat homologs for the above-mentioned proteins, which were identified as potential lymphoma biomarkers in humans. This approach revealed that, apart from FSIP2 (66% identity between cats vs. humans) and IGSF10 (approx. 78.5% identity), all other tested proteins exhibited over 80% similarity (many of them >90%) of sequences between cats and humans (see Table 4 for detailed results). It seems likely that these similar proteins could possibly be used for molecular diagnostics of feline AL. Even though such bioinformatic data need to be first solidly supplemented with wet-lab results, before they can be routinely used in clinical practice, the presented list of protein homologs may constitute the first step towards the development of a molecular diagnostic panel of genes or proteins for AL-afflicted cats.

## 7. Why Does the Number of AL Cases Among Cats Increase, and What About This Disease in Humans?

Although the overall incidence of feline mediastinal and renal lymphomas has declined, likely due to the widespread use of anti-FeLV vaccination, AL has shown the opposite trend. One of the explanations may involve the occurrence of new factor(s) contributing to the increased risk of AL. The previously described negative association between FeLV infection and AL suggests that FeLV does not play a significant role in the pathogenesis of this lymphoma subtype. The divergent correlation between the prevalence of FeLV and the risk of developing different lymphomas highlights the need to investigate alternative etiological agents underlying the rising incidence of AL in cats [7,10]. Another reason for elevated AL morbidity is likely the broader availability of endoscopy and histopathology. Therefore, more AL cases are being accurately identified rather than overlooked. In the literature, it was also reported that inflammation and chronic immune stimulation might be the cause of lymphoma development in cats [65]. Still, that does not explain why AL in cats is so frequent, given that humans are also prone to intestinal inflammation and, additionally, have access to better diagnostic tools. Despite that, human gastrointestinal lymphomas are rare diseases with an incidence rate of one per 1,000,000 inhabitants per year [43]. As this disease is so uncommon in the human population, it is hard to gather cohorts large enough for meaningful genetic or immunological comparisons. It seems probable that different molecular backgrounds and species-specific predispositions, undoubtedly, have a very high impact on the risk of AL development. Nevertheless, no studies have evaluated this hypothesis so far. Thus, the question of what underlies the strikingly different predominance of AL in cats and humans still remains unanswered.

## 8. Conclusions

Feline alimentary lymphoma (AL) is a common and clinically challenging malignancy. Despite advances in understanding epidemiology, classification, and treatment outcomes, its molecular landscape remains poorly explored. High-throughput genomic, transcriptomic, epigenomic, proteomic, and metabolomic studies are limited, though recent improvements in feline genome resources offer new opportunities. However, to use them fully and comprehensively, multicenter studies and big cat cohorts combined with integrated multi-omics pipelines are required. Additionally, comparative analyses with human gastrointestinal T-cell lymphomas reveal molecular similarities, highlighting feline AL as a valuable naturally occurring model of the disease. Its detailed molecular characterization is urgently needed to enhance diagnosis, prognosis, and therapeutic strategies, with potential benefits for both veterinary and human oncology.

## Figures and Tables

**Table 2:** Treatment strategies and survival times in feline lymphomas.

Lymphoma Type	Treatment	Survival Time	
LGAL (EATL II, LGITL, SCL)	Prednisolone (glucocorticoids), chlorambucil [10]	19–29 months [10] median: 2.4 years [25] approx. 800–1300 days (Wright [40], according to other researchers) 204–1267 (Wright’s own studies [40])	
HGAL (EATL I)	CHOP [10]	7–10 months [10] median: 1.5 months (for EATL I overall) [25] median 417 days (12–2962 days) [41]	Wright [40] (For LCL overall: HGAL and LGLL) approx. 100–200 days (Wright [40], according to other researchers) 2–183 (Wright’s own studies [40])
LGLL (EATL I subtype)	CHOP [10]	17: days (median) [10]	

**Table 4:** Human vs. cat comparison of protein sequences.

Protein	Similarity Percentage (Approx.)
TET2	82%
JAK1	97%
JAK3	92%
STAT3	100%
STAT5B	97.50%
KCNB2	95.50%
TP53	81%
CSMD2	92%
CSMD3	94%
IGSF10	78.50%
FSIP2	66%
SETD2	91.50%
NOTCH1	90%
ABL (ABL1)	92%
GNAI2	100%

## Data Availability

No new data were created or analyzed in this study. Data sharing is not applicable to this article.

## Glossary

AL	Alimentary
CE	Chronic enteropathy
CHOP	Cyclophosphamide, hydroxydaunorubicin, Oncovin, prednisolone
CIE	Chronic inflammatory enteropathy
DLBCL	Diffuse Large B-Cell Lymphoma
EATL	Enteropathy-associated T-cell lymphoma
FCE	Feline chronic enteropathy
FeLV	Feline leukemia virus
FIV	Feline immunodeficiency virus
GI	Gastrointestinal
GI-TLPD	Indolent T-cell lymphoproliferative disorder of the gastrointestinal tract
GI-TNKL	Primary gastrointestinal T-/NK-cell lymphoma
HGAL	High-grade alimentary lymphoma
IBD	Inflammatory bowel disease
IGAL	Intermediate alimentary lymphoma
IHC	Immunohistochemistry
ITCL-NOS	Intestinal T-cell lymphoma, not otherwise specified
LCL	Large-cell lymphoma
LGAL	Low-grade alimentary lymphoma
LGITL	Low-grade intestinal T-cell lymphoma
LGLL	Large granular lymphocyte lymphoma
LPE	Lymphocytic plasmacytic enteritis
MALT	Extranodal marginal zone lymphoma of mucosa-associated lymphoid tissue
MEITL	Monomorphic epitheliotropic intestinal T-cell lymphoma
PARR	PCR for Antigen Receptor Rearrangements
PTCL-NOS	Peripheral T-cell lymphomas, not otherwise specified
SCL	Small-cell lymphoma
TALCL	T-cell anaplastic large-cell lymphoma
TCRBCL	T-cell-rich B-cell lymphoma
